# Validating DNA Extraction Protocols for Bentonite Clay

**DOI:** 10.1128/mSphere.00334-19

**Published:** 2019-10-30

**Authors:** Katja Engel, Sara Coyotzi, Melody A. Vachon, Jennifer R. McKelvie, Josh D. Neufeld

**Affiliations:** a Department of Biology, University of Waterloo, Waterloo, Ontario, Canada; b Nuclear Waste Management Organization, Toronto, Ontario, Canada; National Institute of Advanced Industrial Science and Technology

**Keywords:** Wyoming MX-80, bentonite, clay, bacteria, DNA extraction, casein, phosphate

## Abstract

Bentonite clay is an integral component of the engineered barrier system of deep geological repositories (DGRs) that are planned for the long-term storage of high-level radioactive waste. Although nucleic acid extraction and analysis can provide powerful qualitative and quantitative data reflecting the presence, abundance, and functional potential of microorganisms within DGR materials, extraction of microbial DNA from bentonite clay is challenging due to the low biomass and adsorption of nucleic acids to the charged clay matrix. In this study, we used quantitative PCR, gel fingerprinting, and high-throughput sequencing of 16S rRNA gene amplicons to assess DNA extraction efficiency from natural MX-80 bentonite and the same material “spiked” with Escherichia coli genomic DNA. Extraction protocols were tested without additives and with casein and phosphate as blocking agents. Although we demonstrate improved DNA recovery by blocking agents at relatively high DNA spiking concentrations, at relatively low spiking concentrations, we detected a high proportion of contaminant nucleic acids from blocking agents that masked sample-specific microbial profile data. Because bacterial genomic DNA associated with casein preparations was insufficiently removed by UV treatment, casein is not recommended as an additive for DNA extractions from low-biomass samples. Instead, we recommend a kit-based extraction protocol for bentonite clay without additional blocking agents, as tested here and validated with multiple MX-80 bentonite samples, ensuring relatively high DNA recoveries with minimal contamination.

**IMPORTANCE** Extraction of microbial DNA from MX-80 bentonite is challenging due to low biomass and adsorption of nucleic acid molecules to the charged clay matrix. Blocking agents improve DNA recovery, but their impact on microbial community profiles from low-biomass samples has not been characterized well. In this study, we evaluated the effect of casein and phosphate as blocking agents for quantitative recovery of nucleic acids from MX-80 bentonite. Our data justify a simplified framework for analyzing microbial community DNA associated with swelling MX-80 bentonite samples within the context of a deep geological repository for used nuclear fuel. This study is among the first to demonstrate successful extraction of DNA from Wyoming MX-80 bentonite.

## Abstract (precis)

Extraction of microbial DNA from MX-80 bentonite is challenging due to low biomass and adsorption of nucleic acid molecules to the charged clay matrix. Blocking agents improve DNA recovery, but their impact on microbial community profiles from low-biomass samples has not been characterized well. In this study, we evaluated the effect of casein and phosphate as blocking agents for quantitative recovery of nucleic acids from MX-80 bentonite. Our data justify a simplified framework for analyzing microbial community DNA associated with swelling MX-80 bentonite samples within the context of a deep geological repository for used nuclear fuel. This study is among the first to demonstrate successful extraction of DNA from Wyoming MX-80 bentonite.

## INTRODUCTION

Bentonite clay from the Benton Shale near Rock River (Wyoming) is currently being considered for use within the engineered barrier system of a deep geological repository (DGR) for long-term management of high-level radioactive waste (used nuclear fuel). In the Canadian design, the used nuclear fuel will be stored in steel containers for mechanical stability. These containers will be coated with copper to resist corrosion (1–4). Under anoxic DGR conditions, sulfide is considered the primary concern for copper corrosion potential (5) by either diffusion from the surrounding environment or production by sulfate-reducing bacteria (SRB) in the DGR itself. To prevent microbiologically influenced corrosion, the used nuclear fuel containers will be surrounded by highly compacted bentonite (i.e., smectite/montmorillonite-rich swelling clay). The high swelling pressure and low water activity of bentonite, when saturated, contributes to an environment that greatly reduces the activity and survival of microorganisms, including SRB (6–10). Furthermore, compacted bentonite can reduce the diffusion of sulfide to the copper surface, limiting corrosion of the engineered container (9, 11). Characterizing the microorganisms within bentonite using culture-dependent and culture-independent approaches is an important step for evaluating corrosion potential within DGR design components.

Amplicon-based and “meta-omic” methods (e.g., metagenomics and metatranscriptomics) offer enormous potential for assessing the presence and abundance of microorganisms and evaluating their potential activity within a barrier environment. Although such methods are dependent on the recovery of nucleic acids from the charged clay matrix, the extraction of microbial DNA from bentonite clay is challenging due to its low biomass and ability to adsorb nucleic acids. Whereas culturing methods show the presence of microorganisms in clay materials, DNA extractions have failed using various methods (12–15). Indeed, no or very small amounts of DNA have been recovered even from spiked clay samples (12, 16). Nonetheless, Chi Fru and Athar (17) extracted DNA from MX-80 bentonite using an optimized phenol-chloroform method and generated a clone library for analysis. Lopez-Fernandez and colleagues (18) extracted DNA from Spanish bentonite deposits with up to 96% montmorillonite using a gentle sodium dodecyl sulfate (SDS) lysis method with polyethylene glycol precipitation and a final purification using a silica-based column. More recently, Liu and colleagues (19) extracted DNA from Chinese bentonite deposit with 75% montmorillonite (20) using a silica spin column-based kit, including a bead beating cell lysis step. Although Lopez-Fernandez et al. (18) and Liu et al. (19) used high-throughput sequencing to measure bacterial community composition and diversity, no such data are available for the natural Wyoming MX-80 bentonite being considered for a deep geological repository of used nuclear fuel, and no prior studies validated DNA extraction protocols for use with bentonite clay.

Adsorption of nucleic acids to clay surfaces is primarily dependent on electrostatic forces on the negatively charged surface of montmorillonite clay (21, 22). Cations mediate the nucleic acid-clay complex, and divalent cations are more efficient than monovalent cations in this process (23, 24). Adsorption of DNA to montmorillonite clay surfaces therefore increases with decreasing pH (21, 25). At pH values below 5, protonation of the amino groups associated with nucleic acid bases may increase the attraction between positively charged DNA groups and negatively charged montmorillonite clay surfaces (22). Binding is also affected by the physical properties of the DNA. Linear DNA adsorbs more than supercoiled plasmids, likely due to molecule density and the increased availability of free phosphate groups in linearized DNA (26, 27). Although lower molecular weights reduce binding of DNA to clay, guanine-cytosine content has no detectable influence on adsorption (28).

Numerous DNA extraction methods from clay-rich materials have been published using commercial kits (12, 16, 29–33) and/or phenol-chloroform-based protocols (34–37). Previous studies demonstrate that nucleic acid extraction yields can be increased by using “blocking agents” that prevent DNA binding to clay or desorb it from clay by changing the DNA structure or competing for binding sites. These blocking agents include ATP, bovine serum albumin (BSA), casein, natural and synthetic DNA, RNA, deoxynucleoside triphosphates (dNTPs), ethanol, Ficoll, lactose, NaCl, phosphates, polyvinylpyrrolidone, and skim milk (16, 31, 32, 37–41). Proteins, such as BSA, bind to negatively charged clay surfaces via electrostatic and other interactions, preventing DNA from binding (40). Although casein and skim milk have been used extensively, these additives introduce various concentrations of contaminating DNA (31, 32). Natural and synthetic nucleic acids improve recovery and are potentially preferable to biological materials for minimizing bacterial DNA contaminants (31, 41). Phosphate groups bind competetively with DNA to clay and successfully decrease the amount of nucleic acid adsorbed on clay minerals (16, 28, 37, 38). However, added nucleic acids may outcompete sample-specific signals in shotgun metagenomics and metatranscriptomics, especially for low-biomass samples.

Successful desorption of nucleic acids from clay using blocking agents has been demonstrated previously, but evaluations of the quantitative recovery of nucleic acids at various starting concentrations, and the subsequent impact of those blocking agents on microbial community profiles, have not been characterized well. In this study, we used quantitative PCR, gel fingerprinting, and high-throughput 16S rRNA gene sequencing to assess DNA extraction efficiency from natural MX-80 bentonite, with and without the addition of blocking agents and Escherichia coli genomic DNA. This study is among the first to demonstrate successful extraction of DNA from natural Wyoming MX-80 bentonite samples and is unique in providing an experimental validation for the recommended protocol. Given that bentonite clay is considered a proposed engineered barrier component of DGRs for many countries, the ability to monitor microorganisms within clay samples is critical for experiments that assess microbial growth under DGR-like conditions.

## RESULTS AND DISCUSSION

### Initial characterization of Wyoming MX-80 bentonite.

Prior to assessments of DNA recovery in the presence of blocking agents, we generated baseline data for a representative bentonite sample with both cultivation-dependent and cultivation-independent approaches. The numbers of cultivated aerobic and anaerobic bacteria in the Wyoming MX-80 bentonite samples were 2.7 × 10^2^ and 3.3 × 10^1^ CFU per g (dry weight)^−1^, respectively. Using a most probable number (MPN) method, 3.3 × 10^1^ MPN g (dry weight)^−1^ SRB were detected. Previous studies reported cultivable aerobes ranging from 10^2^ to 10^5^ CFU g (dry weight)^−1^ and anaerobes from 10^1^ to 10^4^ CFU g (dry weight)^−1^ (6, 42, 43). Similar to our results, usually no or low numbers of culturable SRB were detected (6, 42–45). Variations among reported estimates of cultured bacteria in dry MX-80 bentonite are likely due to batch heterogeneity, coupled with production and storage differences. A high number of viable but nonculturable (VBNC) bacteria were shown previously in natural and compacted Wyoming MX-80 bentonite based on phospholipid fatty acid (PLFA) analysis. Under increasing water activity conditions, culturability increased by orders of magnitude (6). Others hypothesized that dry MX-80 bentonite powder extracts water from bacterial cells, leaving them in a desiccated state (7). The revival of SRBs indigenous to MX-80 bentonite was dependent on incubation temperatures above 40°C (45).

**FIG 1:**
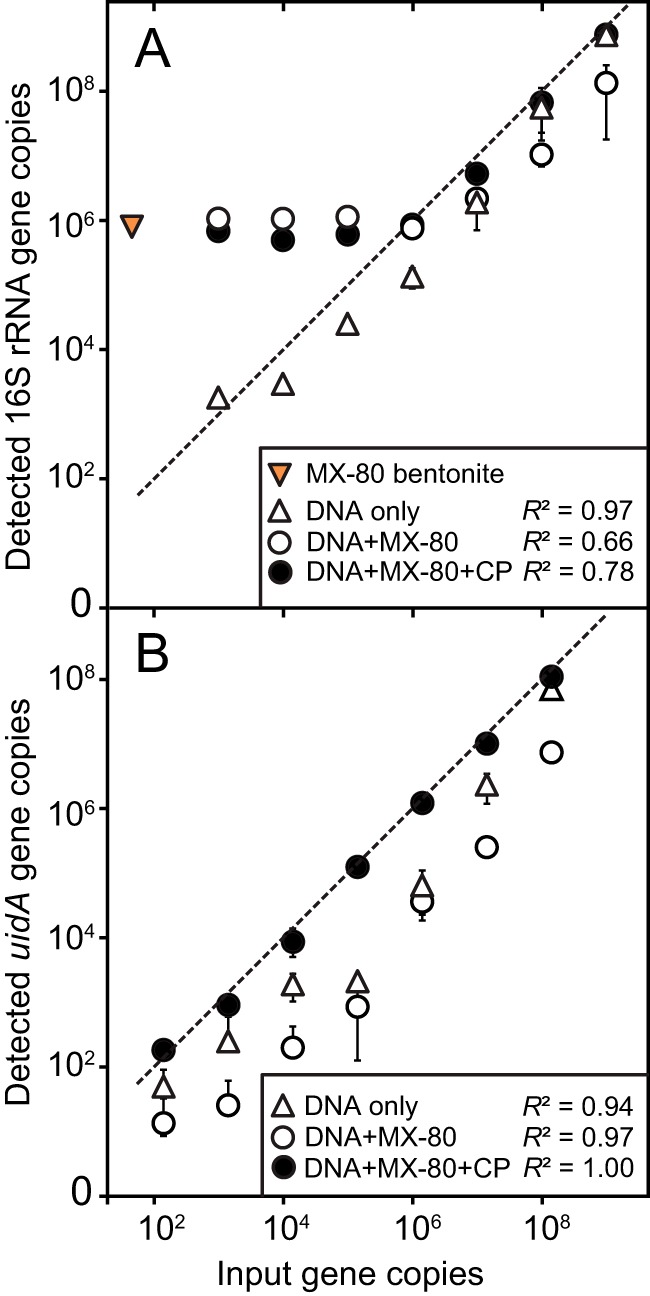
Recovery of DNA from 50 mg (dry weight) MX-80 bentonite spiked with serially diluted Escherichia coli strain W3110 genomic DNA. Recovery was assessed using quantitative PCR targeting bacterial 16S rRNA genes (A) or the single-copy E. coli-specific *uidA* gene (B). Bacterial 16S rRNA genes recovered and quantified from native 50 mg natural MX-80 bentonite (dry weight) is indicated with an orange triangle in panel A. The theoretical optimal DNA recovery (100%) is indicated with dashed lines. Error bars represent standard deviations from two biological replicates.

Genomic DNA extracted from 50 mg (dry weight) natural bentonite using the PowerSoil DNA isolation kit was below the detection limit of the Qubit fluorometric assay but nonetheless yielded 16S rRNA gene amplicons following PCR amplification. Quantitative PCR detected 8.9 × 10^5^ 16S rRNA gene copies in 50 mg MX-80 bentonite (dry weight) (Fig. 1). Due to the swelling of bentonite in the extraction buffer, 50 mg was the maximum amount of sample material that could be used with the PowerSoil DNA isolation kit. To increase DNA yield, we also extracted from 2 g bentonite using the PowerMax DNA isolation kit. In doing so, we obtained 1.3 ± 0.6 ng DNA per g natural MX-80 bentonite, as quantified by the Qubit fluorometric assay, which corresponds to 8.7 × 10^4^ 16S rRNA gene copies per 50 mg (dry weight). The amount of DNA recovered based on fluorometric assay or quantitative PCR and their cell abundance estimates were higher by orders of magnitudes than those with the cultivation-based approaches. However, caution should accompany this comparison, because not all microorganisms may grow under the selected conditions and extracellular DNA could result in an overestimated cell abundance through qPCR analysis.

### DNA extraction yield of spiked bentonite samples.

**FIG 2:**
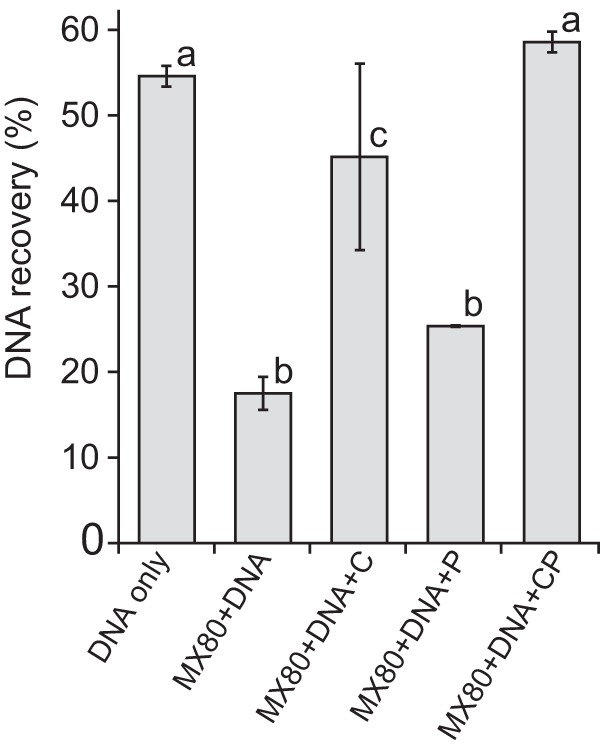
Effect of casein and phosphate (CP) on recovery of Escherichia coli strain W3110 genomic DNA (700 ng) from 50 mg (dry weight) MX-80 bentonite (1:6 slurry) using the PowerSoil DNA isolation kit (Mo Bio). Casein and phosphate were supplemented at 400 mg and 100 μmol per g MX-80 bentonite (dry weight), respectively. DNA was quantified using the Qubit dsDNA HS assay kit (Invitrogen). Error bars represent the standard deviations from duplicates. Different lowercase letters above bars indicate significant differences (Tukey’s honestly significant difference [HSD], *P* < 0.05).

Extraction of microbial DNA from MX-80 bentonite is challenging, presumably due to adsorption of DNA to the charged clay matrix. To examine the efficiency of DNA extraction with the PowerSoil DNA isolation kit (Mo Bio), clay samples were spiked prior to extraction with high-purity genomic DNA from Escherichia coli K-12 strain W3110 of up to 30-kb fragment size (see Table S1 and Fig. S2 in the supplemental material). Without the addition of bentonite clay, the PowerSoil DNA isolation kit recovered 55% of the input DNA (Fig. 2). Recovery was not improved by spiking the DNA after the beating step (Table S1), although the extracted DNA was less sheared (Fig. S2). Kit-associated loss of DNA likely occurred during binding to the silica membrane or subsequent elution, as reported elsewhere (46). When E. coli DNA was incubated with 50 mg MX-80 bentonite (dry weight), recovery decreased by 68% compared to that from the “DNA only” control (Fig. 2), presumably due to adsorption of DNA to MX-80 bentonite. Consistent with this observation, 60% to 80% of chromosomal Bacillus subtilis DNA can be adsorbed to montmorillonite in water (22, 28).

10.1128/mSphere.00334-19.1 FIG S1 The 16S rRNA gene profiles of DNA extractions from natural MX-80 bentonite. Only OTUs at or above 2% relative abundance are shown. DNA was extracted using both the PowerSoil and PowerMax DNA isolation kits (Qiagen). Download FIG S1, PDF file, 0.3 MB. Copyright © 2019 Engel et al. 2019 Engel et al. This content is distributed under the terms of the Creative Commons Attribution 4.0 International license.

10.1128/mSphere.00334-19.2 FIG S2 Pulsed-field gel electrophoresis of Escherichia coli strain W3110 genomic DNA extracted with the PowerMax and PowerSoil DNA isolation kits. M, 1 Kb DNA extension ladder; L, XbaI-cleaved Lambda DNA; 1, PowerSoil extraction from E. coli cells; 2, PowerMax extraction from E. coli cells; 3 and 4: PowerSoil extractions from E. coli genomic DNA (with bead beating) previously extracted with PowerMax kit; 5 and 6: PowerSoil extractions from E. coli genomic DNA (without bead beating) previously extracted with PowerMax kit. Download FIG S2, PDF file, 2.3 MB. Copyright © 2019 Engel et al. 2019 Engel et al. This content is distributed under the terms of the Creative Commons Attribution 4.0 International license.

10.1128/mSphere.00334-19.7 TABLE S1 Recovery of Escherichia coli genomic DNA using the PowerSoil DNA isolation kit (Mo Bio). DNA was quantified using Qubit dsDNA HS assay kit. The standard errors of the means from triplicates are shown. Spiking DNA was added to extractions before the bead beading (+BB) or after the bead beating (−BB) step. Download Table S1, PDF file, 0.1 MB. Copyright © 2019 Engel et al. 2019 Engel et al. This content is distributed under the terms of the Creative Commons Attribution 4.0 International license.

### Influence of blocking agents on DNA extraction yield.

To examine the effect of casein and phosphate on DNA extraction yield, we first tested a relatively high spiking concentration of 0.7 to 1 μg genomic DNA per 50 mg MX-80 bentonite (dry weight). The addition of 10, 50, or 100 μmol phosphate per g MX-80 bentonite (dry weight) did not increase the recovery of DNA significantly compared to that of the spiked MX-80 bentonite control (see Fig. S3A). Concentrations of >100 μmol phosphate decreased DNA recovery (Fig. S3), presumably due to excess phosphates competing for binding to the silica-based membrane; phosphate-silanol interactions are important for double-stranded DNA binding (47). Although previous studies showed that phosphates desorb DNA from clay-rich materials (37, 38), our tested concentrations did not improve recovery with the PowerSoil DNA isolation kit. When Direito et al. (16) substituted the bead beating buffer of the PowerSoil DNA isolation kit with 1 M phosphate, they detected only a small increase in yield when extracting from a spiked montmorillonite sample, likely because phosphates were already contained within the proprietary kit solutions. Considering the competitive interactions between phosphate and DNA for binding to the silica membrane, the ratio of phosphate to clay material is essential for a successful extraction. Any excess phosphate not bound by clay might prevent DNA from binding to the silica membrane and reduce recovery (Fig. S3B). Because the ideal ratio of phosphate to clay will change based on clay type and concentration, phosphate might not be a universal choice for silica membrane-based extraction protocols.

10.1128/mSphere.00334-19.3 FIG S3 Effect of phosphate (P) and casein (C) concentrations on recovery of Escherichia coli strain W3110 genomic DNA. (A) Effect of low phosphate concentrations on recovery of 1 μg genomic DNA from 50 mg MX-80 bentonite (dry weight) using the PowerSoil DNA isolation kit. Phosphate was tested at 10 (P10), 50 (P50), and 100 (P100) μmol PO_4_^3−^ per g MX-80 bentonite (dry weight). (B) Effect of high phosphate concentrations on recovery of 800 ng genomic DNA from 50 mg MX-80 bentonite (dry weight) using the PowerSoil DNA isolation kit. Phosphate was tested at 100 (P100), 500 (P500), and 1,000 (P1000) μmol PO_4_^3−^ per g MX-80 bentonite (dry weight). (C) Effect of casein on recovery of 700 ng genomic DNA from 50 mg MX-80 bentonite (dry weight) using the PowerSoil DNA isolation kit. Casein was tested at 40 (C40) and 400 (C400) mg per g MX-80 bentonite (dry weight). DNA was quantified using Qubit dsDNA HS assay kit. Error bars represent the standard deviations from duplicates. Download FIG S3, PDF file, 0.1 MB. Copyright © 2019 Engel et al. 2019 Engel et al. This content is distributed under the terms of the Creative Commons Attribution 4.0 International license.

The addition of 400 mg casein per g MX-80 bentonite (dry weight) increased the DNA yield to almost DNA-only control levels (Fig. 2). The application of casein at 40 mg per g MX-80 bentonite (dry weight) did not show the same effect (Fig. S3C). The addition of both 400 mg casein and 100 μmol phosphate (CP) recovered DNA-only control levels of DNA (Fig. 2). The proportional increased DNA yield in the presence of casein was in agreement with previous publications (31, 39).

We also tested a phenol-chloroform-based extraction method modified from Lever and colleagues (37) on spiked bentonite samples and found a higher DNA recovery than with the commercial kit (see Fig. S4). The importance of pyrophosphate as a blocking agent for clay-based extractions was apparent, because DNA recovered without blocking agent addition was very low (Fig. S4). Previous results indicated that DNA extraction efficiency from environmental samples was higher with phenol-chloroform-based methods than with the commercial kit protocols (37, 48, 49). The choices of DNA extraction method and 16S rRNA gene region influence microbial diversity profiling (50–53), emphasizing the importance of selecting validated and consistent protocols for research projects that require downstream data comparisons. Although DNA recovery was higher with the phenol-chloroform-based extractions, we used the PowerSoil isolation kit for all subsequent extractions to avoid use of organic solvents and increase scalability for automation of extractions from many samples (54, 55).

10.1128/mSphere.00334-19.4 FIG S4 Recovery of Escherichia coli genomic DNA using method LPII. A 1:6 MX-80 bentonite slurry was spiked with 14 ng genomic DNA per mg MX-80 bentonite. DNA was extracted from 1 g spiked MX-80 bentonite (dry weight). LPII recovered 70% DNA in the control (DNA only) but no DNA when mixed with MX-80 bentonite. The addition of 100 μmol phosphate (P) per g MX-80 bentonite (dry weight) increased recovery from spiked MX-80 bentonite samples. DNA was quantified using Qubit dsDNA HS assay kit. Error bars represent the standard deviations from duplicates. Download FIG S4, PDF file, 0.1 MB. Copyright © 2019 Engel et al. 2019 Engel et al. This content is distributed under the terms of the Creative Commons Attribution 4.0 International license.

### DNA extraction efficiency from MX-80 bentonite at various nucleic acid spiking concentrations.

Given that nearly complete recovery of relatively high nucleic acid concentrations from clay was possible using 400 mg casein and 100 μmol phosphate per g MX-80 bentonite (dry weight), we also assessed the efficiency of selected blocking agents at various nucleic acid concentrations using the PowerSoil DNA isolation kit. Genomic E. coli DNA was diluted serially from 10^9^ to 10^3^ 16S rRNA gene copies and then added to 50 mg MX-80 bentonite (dry weight). Total genomic DNA was estimated using quantitative PCR (qPCR) targeting bacterial 16S rRNA genes or E. coli-specific *uidA* genes. Both qPCR analyses showed that additives increased genomic DNA extraction recovery for the first dilution steps (Fig. 1). Although qPCR data targeting 16S rRNA genes for the DNA-only extracts were consistent with the theoretical optimal DNA recoveries, extracts with MX-80 bentonite and MX-80 plus CP no longer responded linearly after the fourth dilution step (Fig. 1A). Because the 16S rRNA gene primers quantify total bacterial DNA, any DNA extracted from additives, kit reagents, and/or MX-80 bentonite will also be quantified. The proportion of DNA from those sources will increase with lower E. coli genomic DNA concentrations and exceed concentrations of the spiking DNA. Indeed, we detected 8.9 × 10^5^ 16S rRNA gene copies in 50 mg MX-80 bentonite (dry weight). Thus, we conclude that MX-80 bentonite-derived DNA dominated the four highest genomic DNA spiking dilutions (i.e., the four lowest spiking DNA concentrations) (Fig. 1A).

To circumvent clay-specific DNA quantification, we targeted the *uidA* gene specific to E. coli templates. The qPCR data show that additives improved recovery even at the lowest spiking concentrations (Fig. 1B). Although recovery for DNA-only controls and DNA plus MX-80 plus CP was expected to be similar based on results from high spiking concentrations (Fig. 2), extractions with additives had higher recovery for DNA-only treatments at most spiking concentrations. Milk and casein can be contaminated with up to 30% E. coli (56); however, no *uidA* genes were detected in casein and MX-80 bentonite extracts alone (data not shown). Others have reported that casein can increase PCR efficiency (57) but this effect was not seen in our 16S rRNA gene qPCR assay (Fig. 1A). Thus, it is unclear why the DNA-only amplification yields were lower than for DNA plus MX‑80 plus CP treatment samples.

**FIG 3:**
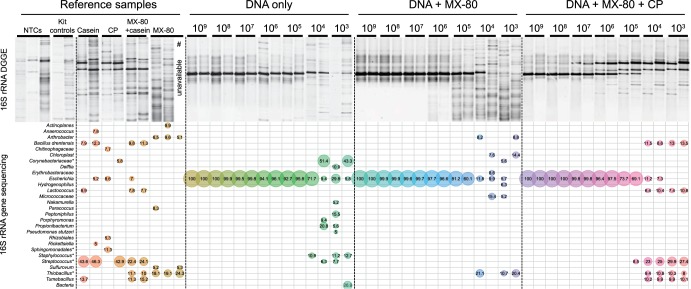
Microbial community profiles of spiked and natural MX-80 bentonite samples. Denaturing gradient gel electrophoresis (DGGE) profiles of the V3 regions of the bacterial 16S rRNA genes at various spiking concentrations (top) and taxonomic distributions in each sample based on high-throughput V3-V4 amplicon sequencing of 16S rRNA genes (bottom). Only operational taxonomic units (OTUs) at or above 5% relative abundance are shown. The OTUs with same classification were pooled and are indicated with asterisks. Casein and phosphate (CP) were added to spiked MX-80 bentonite samples (10^9^ to 10^3^ 16S rRNA gene copies) for adsorption prevention. DNA was extracted from all samples using the PowerSoil DNA isolation kit, except one sample of natural MX-80 bentonite was also extracted with the PowerMax DNA isolation kit (indicated by #).

Both denaturing gradient gel electrophoresis (DGGE) and high-throughput sequencing of 16S rRNA genes demonstrated that, as expected, a single DGGE band dominated extracts from all MX-80 bentonite samples that were spiked with a serial dilution of E. coli genomic DNA (Fig. 3). Consistent with qPCR data of MX-80 bentonite extracts showing ∼10^5^ bacterial template copies per 50 mg (dry weight), “background” MX-80 bentonite DGGE patterns and 16S rRNA gene amplicon sequencing profiles were visible when ∼10^5^ copies of E. coli genomic DNA, or less, were spiked prior to DNA extraction. In addition to demonstrating quantitative and qualitative consistency when generating sample-specific profiles, we here demonstrate reproducible MX-80 bentonite microbial profiles. Although cultivation-based approaches are commonly used for characterizing montmorillonite clay (12, 42, 45), here, we demonstrate that molecular approaches can be effective with a high number of PCR cycles and careful assessment of reagent and kit controls. In addition, our results provide a proof-of-principle methodological approach for assessing the amount of spiked control DNA that would be required to generate quantitative data for 16S rRNA gene sequencing, as proposed recently for use with soil microbial survey work (58).

### Evaluation of reagent and laboratory contaminants.

A total of 4 DNA extraction kit controls and 10 no-template controls were included in high-throughput amplicon sequencing, even though no amplification was detected using gel electrophoresis. Nested no-template controls (NTCs) that were prepared in single tubes (NTC1) had only an average of 73 reads (see Table S2). The NTCs that were included within the 96-well sample plate (NTC2) had higher average read counts (1,586 reads), presumably a result of well-to-well contamination as demonstrated previously (59, 60). However, read counts for NTCs were still relatively low. All DNA extraction kit controls were located within the 96-well plate and had average read counts similar to those of NTC2 (1,585 reads). No common taxa were identified in all controls, except for an operational taxonomic unit (OTU) affiliated with *Escherichia* in NTC2 and kit controls (see Fig. S5), likely arising from well-to-well contamination. The presence of this OTU in NTC1 may be caused by cross-contamination of indexed primers, mixed clusters on the flow cell, and demultiplexing error; however, very low read counts for NTC1 (15 to 214 reads) suggests that the error rate was negligible. At relatively low spiking concentrations (10^4^ and 10^3^ 16S rRNA gene copies) of the DNA-only control, only 29.2% of reads were associated with the dominant *Escherichia*-associated OTU, on average, and most reads were associated with common reagent and laboratory contaminants such as *Corynebacteriaceae*, *Staphylococcus*, and *Delftia* (Fig. 3). We demonstrated previously that low template concentrations affect the 16S rRNA gene profile reproducibility (61). In addition, when Lazarevic et al. (62) extracted DNA from 10^5^ and 10^4^ cells using a spin column kit, only 27.5% to 37.5% of reads were related to the input strains. Salter et al. (63) reported that 16S rRNA gene sequencing of extracts from 10^3^ cells were dominated by contaminants and only 5% to 30% of reads were sample specific. As highlighted previously (63, 64), quantifying sample microbial biomass can help gauge contamination risk. Because our qPCR data indicated that MX-80 bentonite extracts contained ∼10^5^ clay-specific 16S rRNA gene copies per 50 mg (dry weight) of sample, we expected a low proportion of detectable reagent contamination relative to clay-specific sequences.

10.1128/mSphere.00334-19.5 FIG S5 The 16S rRNA gene profiles of DNA extraction kit and no-template controls (NTC) after PANDAseq paired-end read assembly. The PCRs for NTC1 were performed in single tubes, and NTC2 was located within the 96-well plate. PCRs for DNA extraction kit controls (KitCtrl) were also located within the 96-well plate. The plot is based on unrarefied data, and paired-end read counts are shown at the end of the sample name. Only OTUs at or above 5% relative abundance are shown. Download FIG S5, PDF file, 0.2 MB. Copyright © 2019 Engel et al. 2019 Engel et al. This content is distributed under the terms of the Creative Commons Attribution 4.0 International license.

10.1128/mSphere.00334-19.8 TABLE S2 High-throughput sequencing read counts of DNA extraction kit and nontemplate controls (NTCs) after PANDAseq paired-end read assembly. The PCR for NTC1 was performed in a single tube. NTC2 was located within the PCR 96-well plate. Download Table S2, PDF file, 0.1 MB. Copyright © 2019 Engel et al. 2019 Engel et al. This content is distributed under the terms of the Creative Commons Attribution 4.0 International license.

### Effect of DNA contamination from casein on low spiking concentration samples.

**FIG 4:**
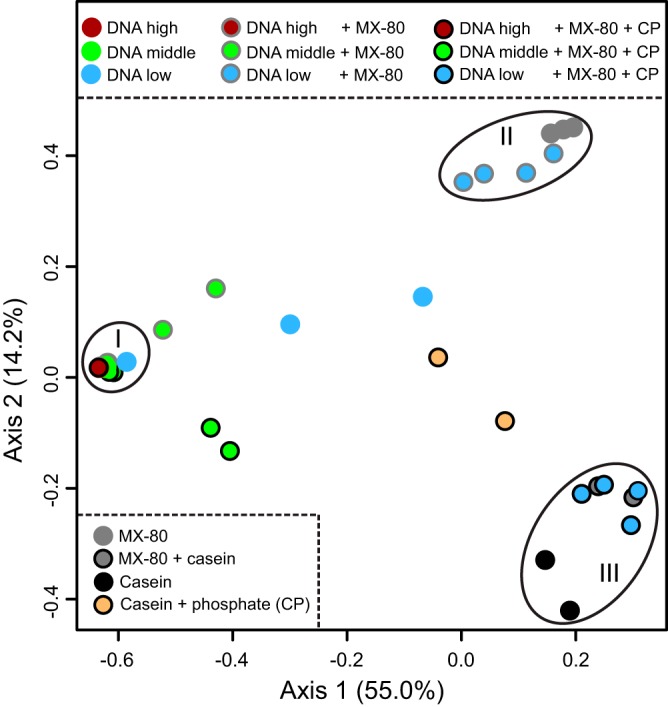
A principal-coordinate analysis (PCoA) ordination plot based on Bray-Curtis distance metrics showing grouping of samples (rarefied to 1,174 reads) based on high spiking concentration (group I), MX-80 bentonite (group II), or the presence of casein (group III). Samples were spiked with either 10^9^ to 10^7^ (DNA high), 10^6^ to 10^5^ (DNA middle), or 10^4^ to 10^3^ (DNA low) 16S rRNA gene copies. The presence of casein and phosphate (CP) in spiked extracts is indicated by black outlines around circles. Most samples with spiking concentrations of >10^5^ are contained within group I. The PCoA ordination plot was generated excluding one replicate of the 10^4^ DNA-only dilution series due to few reads for that sample.

We tested the recovery of DNA from MX-80 bentonite that was spiked with various nucleic acid concentrations. Here, we determined the impact of additives on 16S rRNA gene microbial community profiles using DGGE and high-throughput sequencing. Both DGGE and sequencing approaches detected a high proportion of casein-related contaminant sequences at spiking concentrations of <10^5^ 16S rRNA gene copies per extraction (Fig. 3). Two bands dominated the DGGE profiles of CP extracts, and their presence increased in MX-80 bentonite samples as DNA spiking concentration decreased (Fig. 3, top). Approximately 45% of OTUs in casein extract sequence data were associated with *Streptococcus* (Fig. 3, bottom), and these lactic acid bacteria are prevalent in milk samples (65). In MX-80 bentonite samples with low DNA spiking concentrations (10^4^ and 10^3^ 16S rRNA gene copies), 23% to 30% of OTUs were associated with *Streptococcus*. Other contaminants originating from casein were associated with *Tumebacillus*, Bacillus drentensis, *Lactococcus*, and *Lactobacillus*, accounting for 30% to 37% of reads at low spiking concentrations. On average, only 18% of reads were associated with spiking DNA or the six most abundant OTUs identified in natural MX-80 bentonite. Without additives, 33% of reads were associated with those sample-specific OTUs (supplemental OTU table [see “Data availability” paragraph below]). As mentioned previously, at low spiking concentrations of the DNA-only control, only 29% of reads were associated with the dominant *Escherichia* OTU, and the majority of reads were associated with laboratory and reagent contamination. Despite large proportions of reagent contaminants in samples spiked with 10^4^ and 10^3^ 16S rRNA gene copies, MX-80 bentonite samples nonetheless grouped with extracts from natural MX-80 bentonite when additives were absent (Fig. 4). In the presence of CP, the same samples grouped with casein extracts. Only samples with more than 10^6^ 16S rRNA gene copies grouped distinctly from additive samples.

### DNA extraction protocol verification.

**FIG 5:**
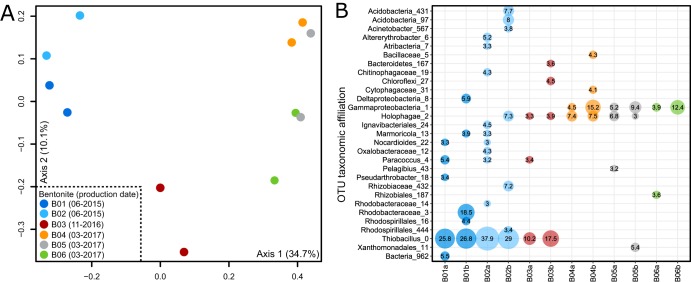
Grouping of natural Wyoming MX-80 bentonite samples based on production dates in a principal-coordinate analysis (PCoA; Bray-Curtis dissimilarity metric) ordination plot (A) and microbial community profiles generated using high-throughput V4-V5 16S rRNA gene amplicon sequencing (B). Only operational taxonomic units (OTUs) at or above 3% relative abundance are shown, and numbers in the bubbles represent the relative abundance (%) of each OTU in the corresponding library. Lowercase a or b after each sample name distinguishes duplicate extractions from the same sample batch. For an additional description of each sample, see Table 1.

We detected increased nucleic acid recoveries from MX-80 bentonite using casein and phosphate, but casein-associated contaminant DNA was detected at low spiking concentrations. Any additive increases the risk of contamination, and we therefore recommend the PowerSoil and PowerMax DNA isolation kits without additional blocking agents for future MX-80 bentonite DNA extractions. To verify the suitability of the extraction protocol for MX-80 bentonite samples, we extracted DNA from five additional Wyoming MX-80 bentonite samples from different production dates (Table 1). The amount of DNA recovered from those samples was consistent, with 1.8 to 2.5 ng per g (dry weight) bentonite (Table 1). High-throughput 16S rRNA gene sequencing of DNA extracted from natural Wyoming MX-80 bentonite samples showed that detected microbial communities differed based on production date (Fig. 5A). The MX-80 bentonite samples produced in June 2015 were associated with 25.8% to 37.9% of the sequences associated with *Thiobacillus* sp. (Fig. 5B), which are likely Gram-negative sulfur-oxidizing bacteria (66). Other batches were characterized by OTUs affiliated with the *Holophagae* (phylum *Acidobacteria*) and members of the *Gammaproteobacteria*.

**TABLE 1:** Summary of analyzed Wyoming MX-80 samples

ID^*a*^	Lot no.	Production date (mo-yr)	DNA (ng/g)
B01	065275768	06-2015	1.3
B02	065275772	06-2015	2.2
B03	116315319	11-2016	2.5
B04	037324182	03-2017	2.2
B05	037324184	03-2017	2.2
B06	037324190	03-2017	1.8

a ID, identifier.

Few previous studies have demonstrated successful recovery of DNA from natural bentonite clay. Although Chi Fru and Athar (17) extracted DNA from Wyoming MX-80 bentonite, they identified primarily *Bacillus* spp. in the associated clone library. Only 3% of reads in our high-throughput sequencing results from all six Wyoming-MX-80 samples were associated with the *Bacillus* genus. Both Lopez-Fernandez et al. (18) and Liu et al. (19) used high-throughput sequencing to assess bacterial diversity in DNA extracts from natural bentonite deposits in Spain and China. Common bacteria, such as those affiliated with the *Acidobacteria* and *Paracoccus*, were identified (18, 67); however, the dominant microorganisms detected were distinct for each deposit.

### Conclusions.

We evaluated the effect of casein and phosphate as blocking agents for quantitative recovery of nucleic acids from MX-80 bentonite at various starting concentrations and detected increased recovery of nucleic acids from bentonite using casein and phosphate (Fig. 1 and 3). However, predominantly casein-associated contaminant DNA was detected at spiking concentrations of <10^5^ 16S rRNA gene copies (Fig. 2). Although we have previously used 30 min of UV light to decontaminate additives successfully (68), this treatment was insufficient to remove background DNA contamination from casein; therefore, casein is not recommended as a blocking agent for DNA extraction from low-biomass clay samples. Synthetic nucleotides as blocking agents can be designed to minimize interference with gene-specific studies, but they might affect downstream analyses such as shotgun metagenomics. Nucleic acid contamination in any blocking agent will reduce sample-specific signal in low-biomass samples, and decontamination might not remove all contaminants or might negatively affect PCR efficiency (69, 70). Reagent and laboratory contaminations are inevitable and require extensive postrun analysis, as demonstrated here and elsewhere (59).

Because any additive increases the risk of further contamination, we recommend the PowerSoil and PowerMax DNA isolation kits without additional blocking agents for future MX-80 bentonite DNA extractions. Indeed, the high sensitivity of PCR with the equivalent of 50 cycles and subsequent fingerprinting or high-throughput sequencing analyses suggest that some additional loss of DNA is a reasonable trade-off in order to minimize methodological customization and the potential for introducing additional sources of contamination. We successfully extracted DNA from six Wyoming MX-80 bentonite samples and generated high-throughput 16S rRNA gene profiles. Together, our results provide a simplified framework for analyzing microbial community DNA associated with swelling MX-80 bentonite samples within the context of a DGR for used nuclear fuel. Although transcriptomics would focus on active members of the community in bentonite clays, it will be very challenging to extract RNA successfully from Wyoming MX-80 bentonite.

## MATERIALS AND METHODS

### Extraction of genomic E. coli DNA and copy number calculation.

Escherichia coli K-12 strain W3110 was grown in 200 ml LB medium overnight at 37°C and 180 rpm. Cells were harvested by centrifugation at 7,000 × *g* for 10 min. A 2-g cell pellet was used for nucleic acid extraction with the PowerMax DNA isolation kit (Mo Bio Laboratories, Carlsbad, CA, USA) according to the manufacturer’s instructions. Genomic DNA concentration was determined using the Qubit dsDNA High Sensitivity (HS) assay kit (Invitrogen, Carlsbad, CA, USA) with fluorescence measured on a FilterMax F5 MultiMode plate reader (Molecular Devices, San Jose, CA, USA) at excitation and emission wavelengths of 485 and 525 nm, respectively. The quality of extracted E. coli DNA was assessed using a NanoDrop 2000 spectrophotometer (Thermo Scientific, Waltham, MA, USA) and pulsed-field gel electrophoresis (PFGE) using a CHEF Mapper XA apparatus (Bio-Rad, Hercules, CA, USA). The PFGE gel was run at 14°C for 16 h at 5.5 V/cm with a 1- to 6-s linear pulse in a 1% agarose gel. The gels were stained using GelRed (Biotium, Fremont, CA, USA) and visualized with an AlphaImager HP System (ProteinSimple, San Jose, CA, USA).

The spike samples of genomic E. coli DNA were prepared as 10-fold serial dilutions ranging from 9.8 × 10^8^ to 9.8 × 10^2^ 16S rRNA gene copies per extraction using E. coli K-12 strain W3110 genome size (71) and the following equation (72): 16S rRNA gene copies = 7 operons × (DNA amount [g] × 6.02 × 10^23^ [copies/mol])/(4.65 × 10^6^ bp × 650 g/mol/bp). For simplicity, 9.8 × 10^8^ to 9.8 × 10^2^ 16S rRNA gene copies are reported as 10^9^ to 10^3^ throughout, respectively.

### Wyoming MX-80 bentonite.

Six Wyoming MX-80 bentonite samples from different production dates were analyzed in this research (Table 1). The MX-80 bentonite sample B01 was used for the spiking experiments. This sample was processed on 3 June 2015 (lot 065275768) by Caldic Canada (Mississauga, ON, Canada). Moisture content and pH were determined by Caldic Canada as 18.5% and 9.96, respectively. The MX-80 bentonite consists of approximately 74% to 90% montmorillonite (73, 74), causing swelling on water uptake. The swelling index was determined with 28 ml/2 g (ASTM D5890-06) for oven-dried (24 h at 105°C) bentonite.

### Enumeration of naturally occurring bacteria in Wyoming MX-80 bentonite.

Enumeration of cultivable bacteria in bentonite was conducted as previously described (6), with several modifications. Bentonite suspensions were prepared by slowly adding 2 g bentonite powder (18.5% moisture content) to 18 ml liquid medium while it was continuously agitated using a vortex to avoid clumping. The bentonite suspension was further mixed in a rotating incubator at 15 rpm for 30 min at room temperature. For the enumeration of cultivable aerobic and anaerobic bacteria, the bentonite suspension and 10-fold serial dilutions were prepared in R2A medium (M1687; HiMedia Laboratories, West Chester, PA, USA). Serial dilutions were plated on R2A agar plates, in triplicates, and incubated under oxic or anoxic conditions at 30°C for 7 or 28 days, respectively. A 5-tube most probable number (MPN) method was used for the enumeration of cultivable sulfate-reducing bacteria incubated under anoxic conditions at 30°C for 4 weeks. The bentonite suspension and 10-fold serial dilutions were prepared in media. Sulfate-reducing bacteria were quantified in Triple Pack medium (M803; HiMedia Laboratories) and assessed for black ferrous sulfide (FeS) precipitation, signifying hydrogen sulfide production and, consequently, sulfate reduction.

### Large-scale DNA extraction from natural Wyoming MX-80 bentonite.

Total genomic DNA from natural bentonite powder was extracted using the PowerMax DNA isolation kit (Mo Bio Laboratories). A total of 2 g of bentonite powder was slowly added to 15 ml PowerBead solution while being agitated. After addition of lysis solution, the tube was incubated at 65°C for 30 min before bead beading for 10 min at 30 Hz (Mixer Mill MM 400; Retsch, Germany). The remainder of the extraction was carried out according to the manufacturer’s instructions. Purified DNA was eluted in 2 ml of 10 mM Tris. Nucleic acids were precipitated using 4 μl/ml Co-Precipitant linear polyacrylamide (Bioline, Germany), 0.1 volumes of 5 M NaCl (prepared in 0.2-μm filter-sterilized PCR water [HyClone HyPure Water]; GE Healthcare Life Sciences, Logan, UT, USA), 1 volume of isopropanol (high-pressure liquid chromatography [HPLC] grade), and stored at −20°C overnight. Precipitated DNA was pelleted by centrifugation at 13,000 × *g* for 30 min and then washed with 80% ethanol (HPLC grade), air dried, and eluted in 150 μl of 10 mM Tris. Aliquots were frozen at −20°C until PCR analysis. A control extraction without any sample was carried out in parallel to assess potential contamination from kit reagents.

### DNA extraction from natural and spiked Wyoming MX-80 bentonite.

Genomic DNA was extracted from natural and spiked MX-80 bentonite samples using the PowerSoil DNA isolation kit (Mo Bio Laboratories). A control extraction without any sample was carried out in parallel to assess reagent contamination. Powdered MX-80 bentonite was used to prepare a 1:6 slurry (solid/liquid ratio) in sterile nuclease and nucleic acid-free PCR water (GE Healthcare Life Sciences). As described above, bentonite suspensions were prepared by slowly adding bentonite powder to liquid with vortex agitation to avoid clumping. The MX-80 slurry was spiked with E. coli DNA at various concentrations, mixed by inversion, and incubated in a rotating incubator at 15 rpm for 30 min at room temperature to enable DNA binding to the clay matrix; note that we did not assess the specific proportion of DNA adsorbed to MX-80 bentonite. Genomic DNA extractions from 0.3-ml slurries (50 mg MX-80 bentonite [dry weight]) were performed in duplicates according to the manufacturer’s instructions with several modifications. Specifically, after the addition of lysis buffer, samples were incubated at 65°C for 30 min to enhance lysis and desorption of DNA from clay matrix. Bead beating was conducted in the FastPrep-24 instrument (MP Biomedicals, Solon, OH, USA) at 5.5 m/s for 45 s. All supernatant was transferred at every step for maximum nucleic acid recovery. Total DNA was quantified using a Qubit dsDNA High Sensitivity assay kit (Invitrogen).

### Blocking agents.

To assess a representative subset of many possible blocking agents commonly used in DNA extractions, we tested casein (catalog number C7078; 400 mg per g MX-80 bentonite [dry weight]; Sigma) and sodium pyrophosphate (catalog number S6422; 100 μmol PO_4_^3−^ per g MX-80 bentonite [dry weight], 0.4 M; Sigma) for our bentonite clay nucleic acid extractions. Because Ikeda and colleagues (31) tested both casein and BSA on DNA extractions of Andisol soil samples and found that casein was successful in extracting DNA from more Andisol soil samples than BSA, we selected casein as a representative protein-based blocking agent for this study. To remove potential nucleic acid contamination from casein, as reported previously (31), we added casein (20 mg) to the Mo Bio bead beating solution and UV treated the tube for 30 min on a 302-nm UV transilluminator. Phosphate was prepared in PCR water and UV treated in the same way. We chose UV illumination as the method for decontamination, because we have used it successfully to remove contaminants from additives in DNA extractions (68). We tested casein and phosphates on spiked and natural MX-80 bentonite samples with the PowerSoil DNA isolation kit using the protocol and modifications described above. We did not test the use of nucleic acids as blocking agents, because they would coextract with low-biomass target DNA and influence downstream applications, such as metagenomics. It was shown by Jacobsen and colleagues (41) that a commercially available blocking reagent increased the yield from low-biomass clay subsoil without introducing contaminating DNA. However, we have not tested this blocking agent in this research.

### Denaturing gradient gel electrophoresis analysis.

The V3 regions of bacterial 16S rRNA genes were amplified using primer pair 341F-GC and 518R (75). The 50-μl PCR mix contained 1 × ThermoPol buffer, 0.2 μM each primer, 200 μM dNTPs, 30 μg BSA, 1.25 U *Taq* DNA polymerase (New England Biolabs, Ipswich, MA, USA), and 2 μl of template. The PCR was performed in two rounds by adding 1 μl of amplification product from the first PCR (PCR1) into the second PCR (PCR2). The PCR amplifications were performed as follows: 95°C for 3 min, 35 (PCR1) or 15 (PCR2) cycles of 95°C for 15 s, 55°C for 30 s, and 68°C for 30 s, and a final extension of 68°C for 7 min. Equal amounts of PCR amplicons were separated on a 10% (wt/vol) polyacrylamide gel with a denaturant gradient ranging from 30% to 70% to a maximum of 20 μl for samples with low PCR yield. The gels were run as previously described (76) for 15 h at 85 V in a DGGEK-2401 denaturing gradient gel electrophoresis (DGGE) system (C.B.S. Scientific Company, San Diego, CA, USA), stained with SYBR green I DNA stain (Invitrogen) for 1 h, and scanned using the Molecular Imager Pharos FX Plus (Bio-Rad).

### Quantitative PCR.

Genomic DNA in spiked and natural MX-80 bentonite extracts was quantified by targeting multicopy bacterial 16S rRNA genes using primers 341F/518R and the single-copy E. coli-specific *uidA* gene (coding for β-d-glucuronidase) using primers uidA405F/uidA405R (77). All PCR amplifications were performed in duplicates. For the *uidA* gene PCR, the 15-μl reaction volume contained 1× SsoAdvanced Universal SYBR green Supermix (Bio-Rad), 0.3 μM each primer, 7.5 μg BSA, and 2 μl of template. The 16S rRNA gene PCR was performed in 10-μl reaction volumes with PCR components in concentrations as listed above, without the addition of BSA. The PCRs were performed on a CFX96 Real-Time PCR detection system (Bio-Rad). For *uidA* gene amplification, PCR conditions were 98°C for 3 min followed by 40 cycles of 98°C for 15 s and 60°C for 60 s. For 16S rRNA genes, the PCR conditions were 98°C for 3 min followed by 40 cycles of 98°C for 15 s and 55°C for 30 s. Purified E. coli genomic DNA was used as a standard template for both PCR protocols. Amplification efficiencies ranged from 90.3% to 99.6%, and all coefficients of determination (*R*^2^) exceeded 0.995. Starting DNA copy numbers were calculated from the linear regression equation of each standard curve.

### Amplification of 16S rRNA genes and Illumina sequencing.

The V3-V4 regions of the 16S rRNA genes were amplified using universal prokaryotic dual-indexed primers Pro341F and Pro805R (78). In addition to the unique 6-bp index sequence for sample multiplexing, each primer contained Illumina flow cell binding and sequencing sites (79). The PCR was set up in a PCR workstation using ISO 5 HEPA-filtered air (AirClean Systems, Creedmoor, NC, USA). The surface was cleaned with UltraClean Lab Cleaner (Mo Bio) and treated with UV light irradiation for 15 min. In addition, tubes, 96-well plates, PCR water, and BSA were UV treated on a 302 nm transilluminator (ProteinSimple, San Jose, CA, USA) for 15 min. The 25-μl PCR mixture contained 1 × ThermoPol buffer, 0.2 μM forward primer, 0.2 μM reverse primer, 200 μM dNTPs, 15 μg BSA, 0.625 U *Taq* DNA polymerase (New England Biolabs), and 2 μl of template (up to 10 ng). Each PCR was prepared in triplicates, with two rounds by adding 1 μl product from PCR1 into PCR2. The PCRs were performed as follows: 95°C for 3 min, 35 (PCR1) or 15 (PCR2) cycles of 95°C for 30 s, 55°C for 30 s, and 68°C for 1 min, and a final extension of 68°C for 7 min. Equal quantities of PCR2 amplicons were pooled (average of 10 μl per sample). Twenty microliters of DNA extraction blanks and no-template controls (NTCs) was included, even though no amplicon was visible in a stained agarose gel. The pooled 16S rRNA gene amplicons were excised from an agarose gel and purified using a Wizard SV Gel and PCR Clean-Up system (Promega, Madison, WI, USA). A 5-pM library containing 5% PhiX control library (Illumina, San Diego, CA, USA) was sequenced on a MiSeq instrument (Illumina) using a 2 × 250-cycle MiSeq reagent kit v2 (Illumina Canada, Vancouver, BC, Canada). For comparison of six natural Wyoming MX-80 bentonite samples, the V4-V5 region of 16S rRNA genes was amplified using universal prokaryotic primers 515F-Y (80) and 926R (81) according to the method described above but at an annealing temperature of 50°C.

### Illumina sequence analysis.

All MiSeq reads were demultiplexed using MiSeq Reporter software (version 2.5.0.5; Illumina). Reads were assembled using the paired-end assembler for Illumina sequences (PANDAseq, version 2.8) (82) with a quality threshold of 0.9, 8-nucleotide minimum overlap, and 300-nucleotide minimum assembled read length. Assembled reads were analyzed using Quantitative Insights Into Microbial Ecology (QIIME version 1.9.0) (83), managed by automated exploration of microbial diversity (AXIOME version 1.5) (84). Sequences were clustered using UPARSE algorithm USEARCH version 7.0.1090 (85) at 97% identity and aligned with the Python Nearest Alignment Space Termination tool (PyNAST version 1.2.2) (86). All representative sequences were classified using the Ribosomal Database Project (RDP version 2.2) (87) with a stringent confidence threshold (0.8). Taxonomy was assigned using the SILVA database release 128 (88). Chimeric sequences were filtered with UCHIME (89). Bubble plots showing taxonomy profiles were created using the ggplot2 package (90) in R v.3.4.4 using operational taxonomic unit (OTU) tables generated by AXIOME.

### Data availability.

All sequences were deposited into European Nucleotide Archive (https://www.ebi.ac.uk/ena) with study accession number PRJEB29317. Supplemental OTU tables were deposited at 10.5281/zenodo.3459859 and 10.5281/zenodo.3459870.

10.1128/mSphere.00334-19.6 TEXT S1 DNA extraction from spiked Wyoming MX-80 bentonite using a chloroform-based extraction method. Download Text S1, PDF file, 0.1 MB. Copyright © 2019 Engel et al. 2019 Engel et al. This content is distributed under the terms of the Creative Commons Attribution 4.0 International license.
