# Supplementary figures and images for: Validating DNA Extraction Protocols for Bentonite Clay
Source: mSphere. 2019 Oct 30;4(5):e00334-19. doi: 10.1128/mSphere.00334-19 (PMC6821930; doi:10.1128/mSphere.00334-19)

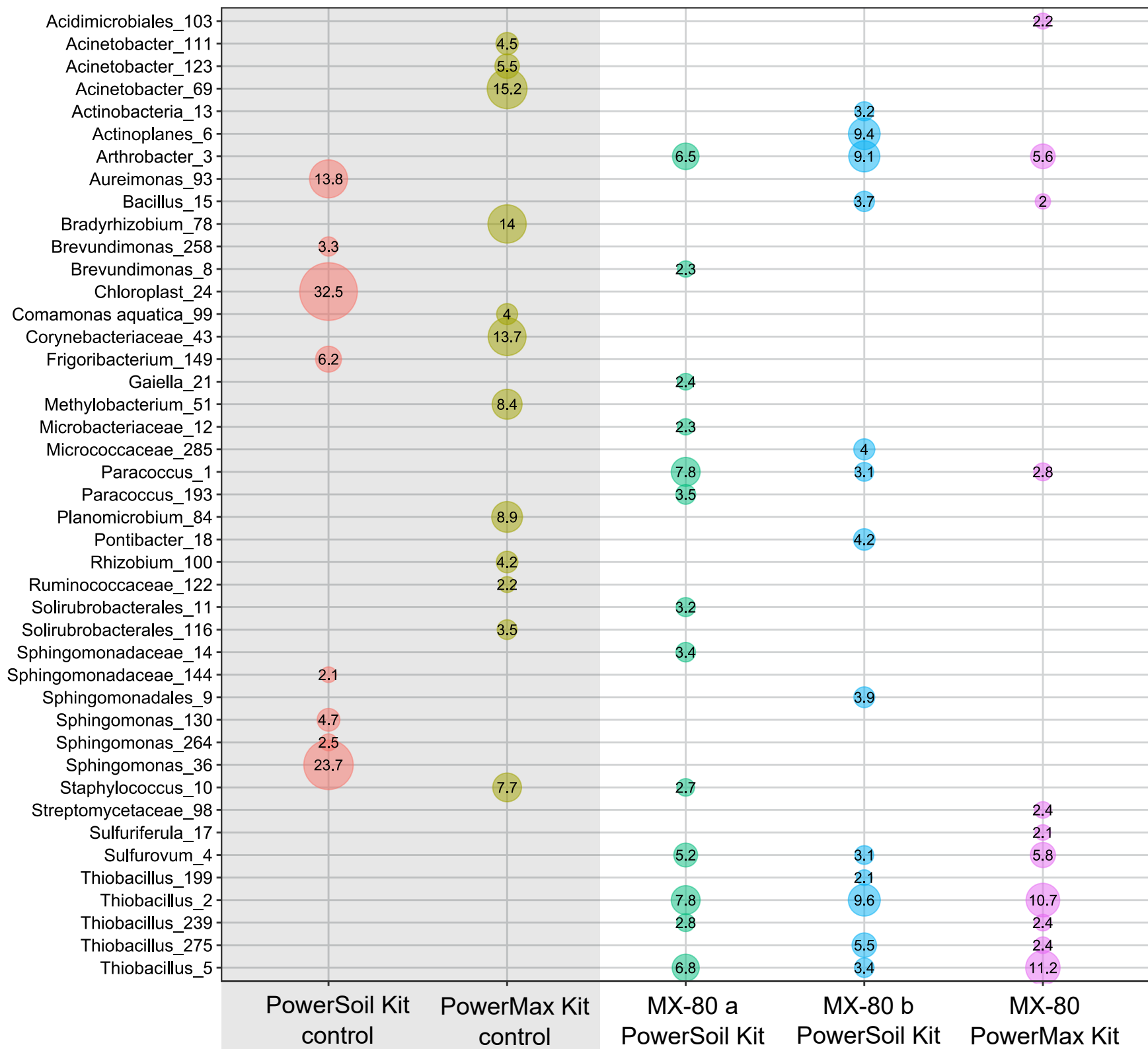

Supplement: FIG S1 [file mSphere.00334-19-sf001.pdf]

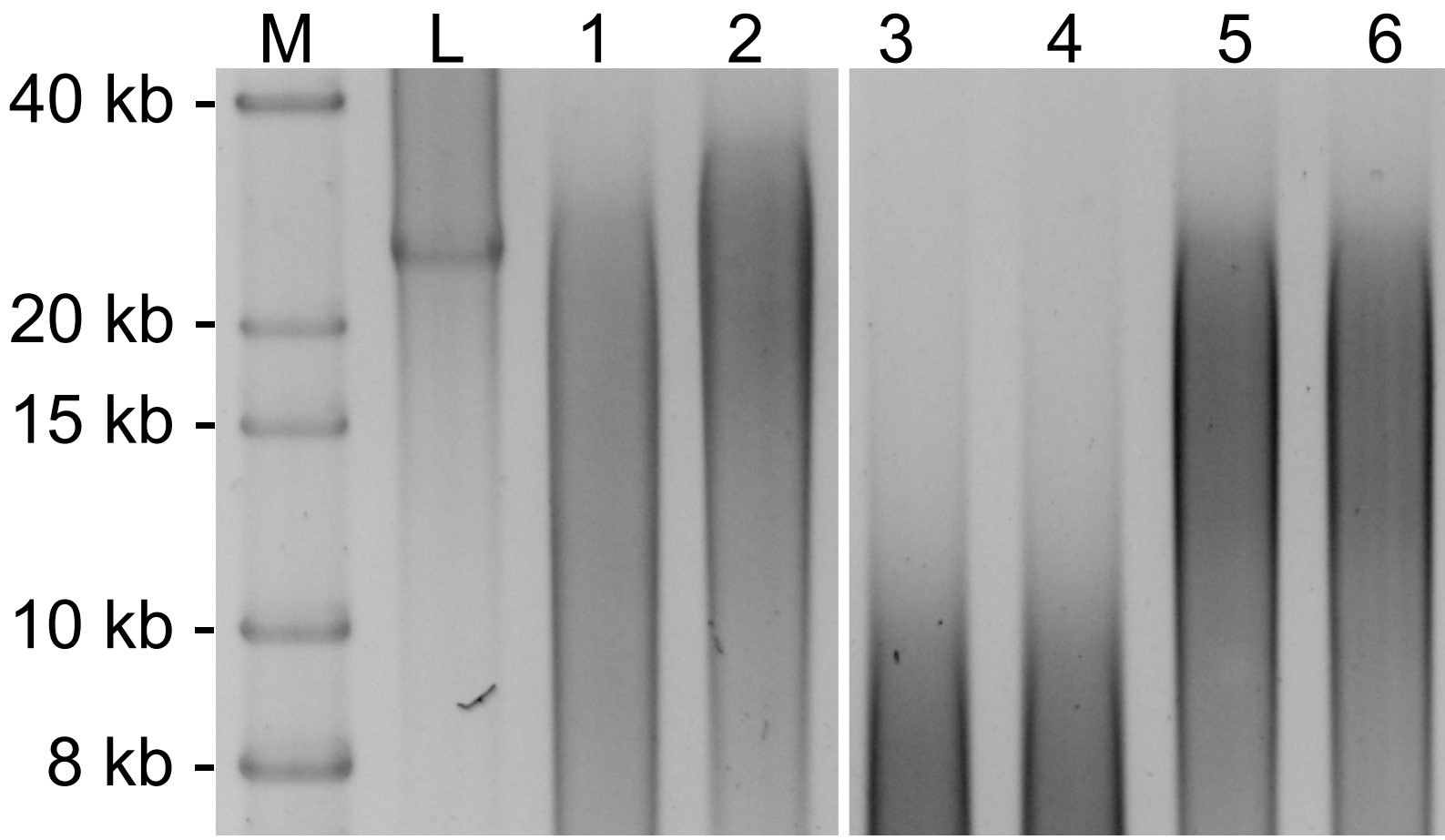

Supplement: FIG S2 [file mSphere.00334-19-sf002.pdf]

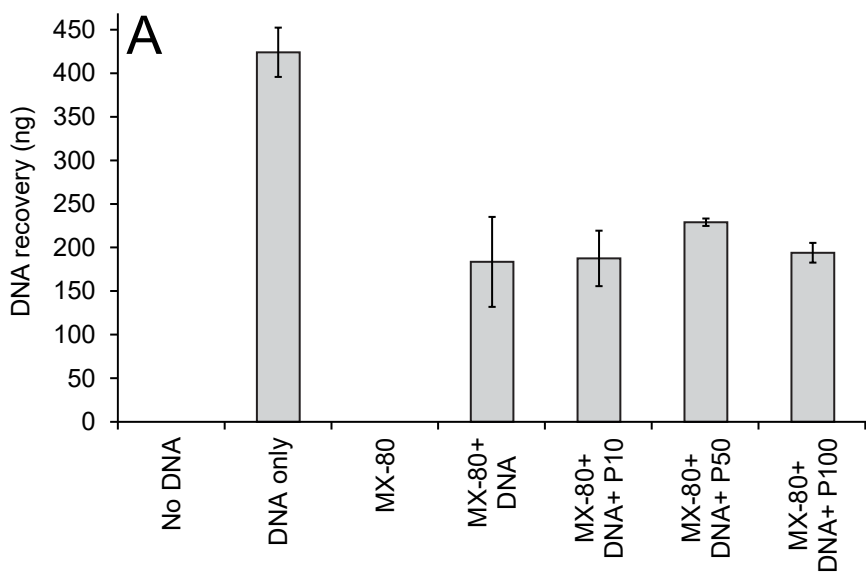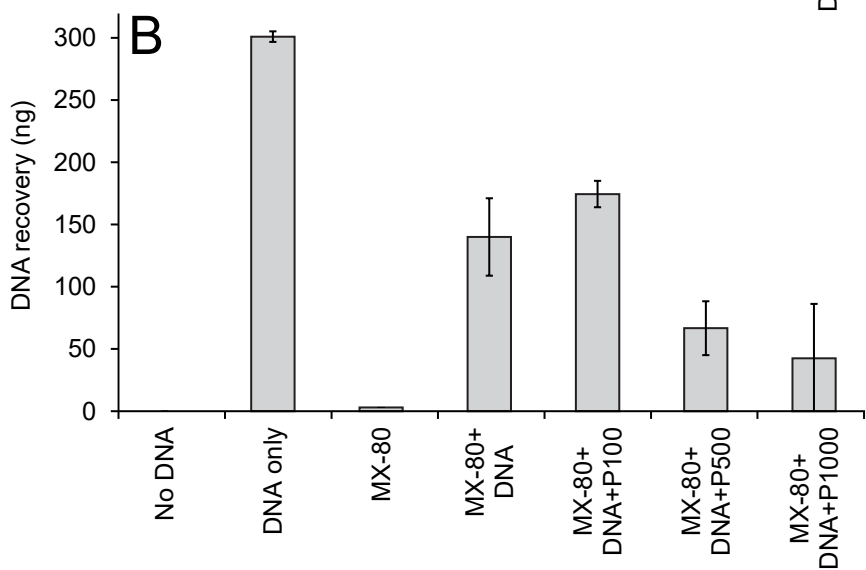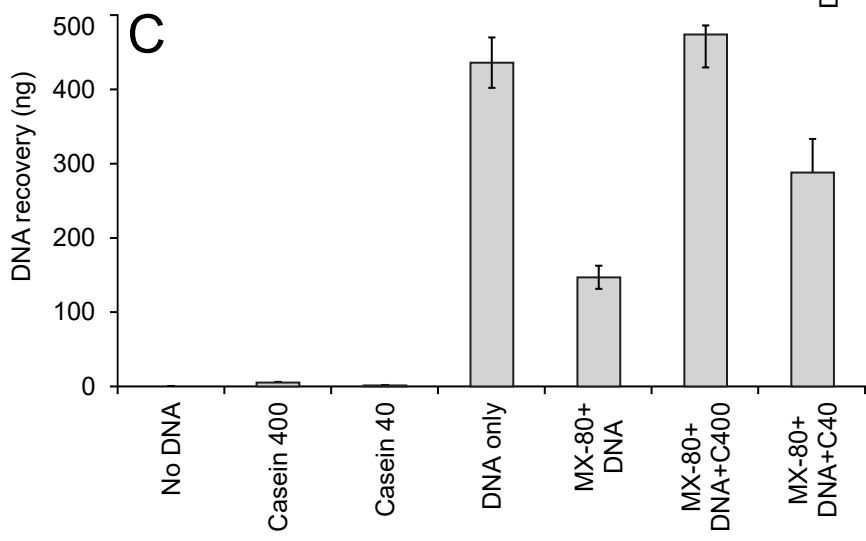

Supplement: FIG S3 [file mSphere.00334-19-sf003.pdf]

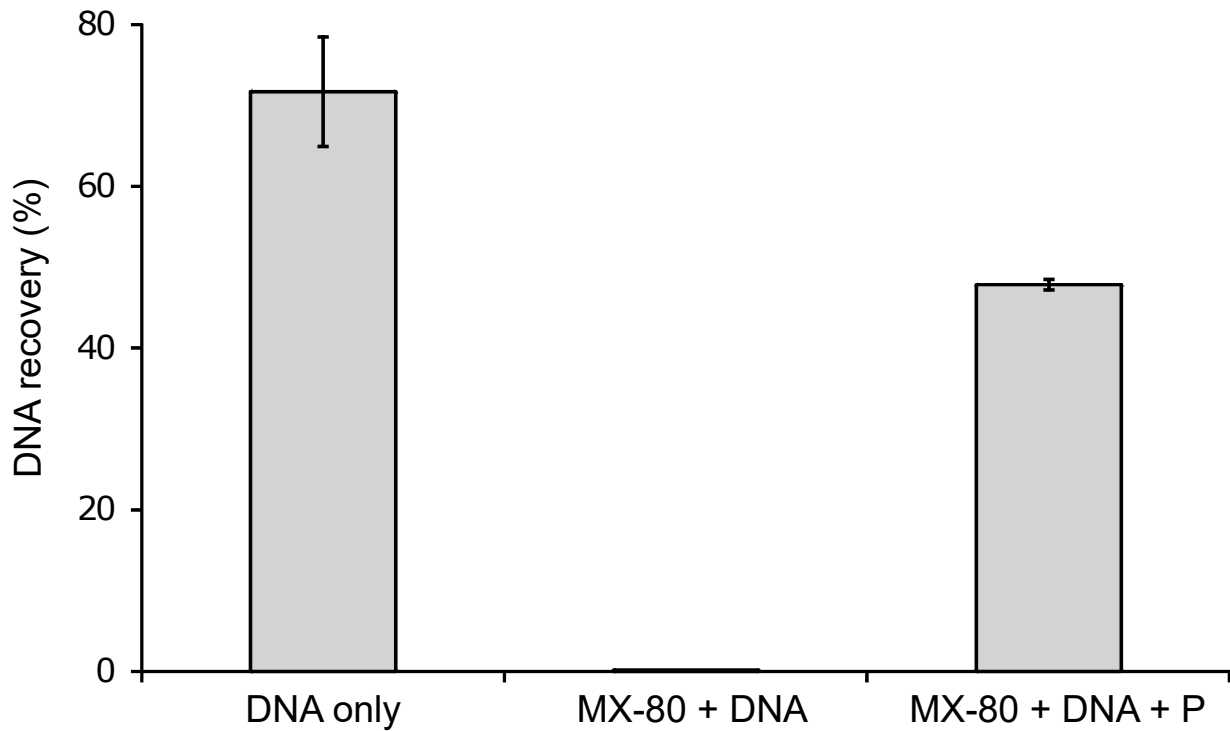

Supplement: FIG S4 [file mSphere.00334-19-sf004.pdf]

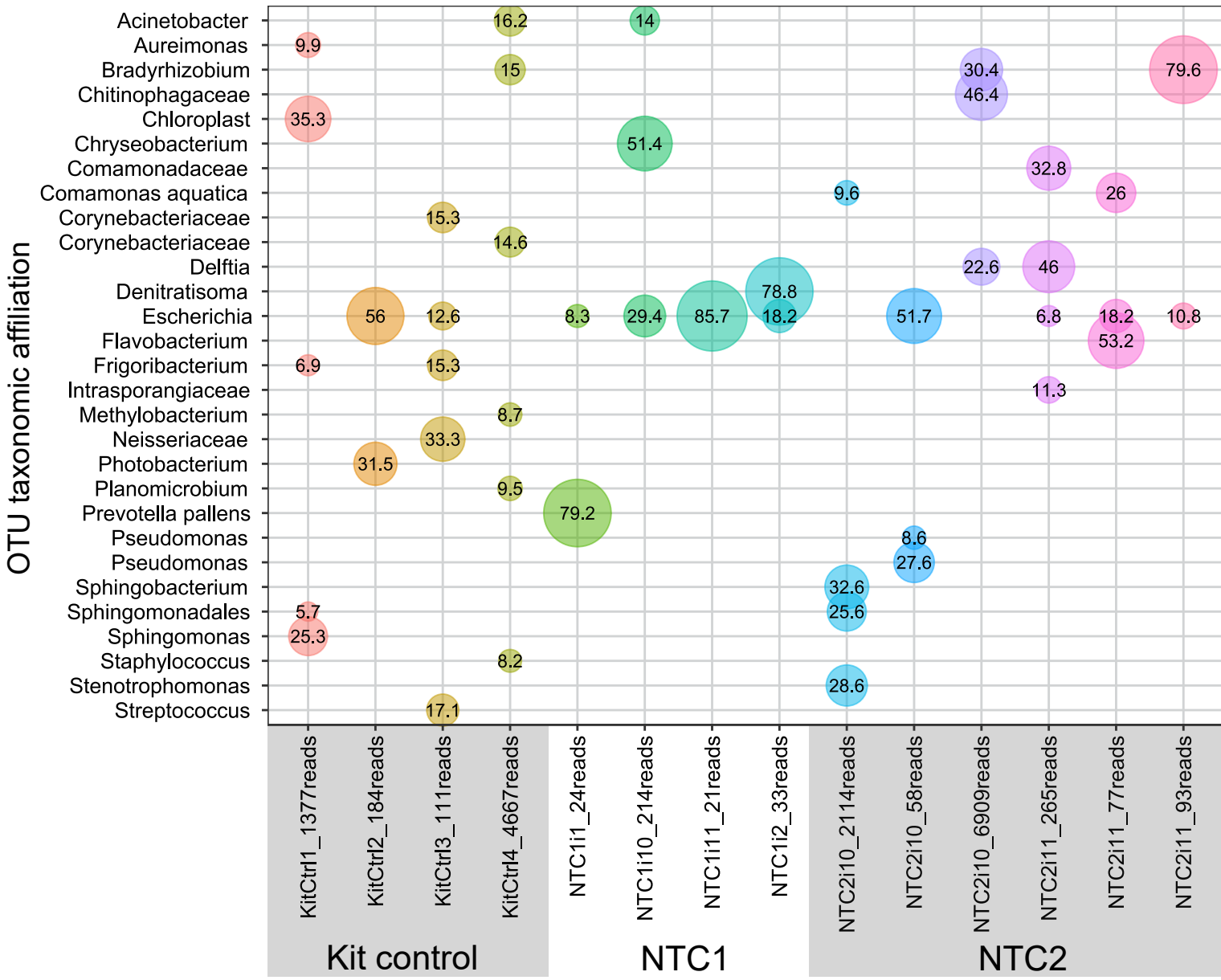

Supplement: FIG S5 [file mSphere.00334-19-sf005.pdf]
