# Supplementary material for: Validating DNA Extraction Protocols for Bentonite Clay
Source: mSphere. 2019 Oct 30;4(5):e00334-19. doi: 10.1128/mSphere.00334-19 (PMC6821930; doi:10.1128/mSphere.00334-19)
Supplement: TABLE S1 [file mSphere.00334-19-st001.pdf]

Extraction	Recovery (ng)	Recovery (%)
MO BIO + BB	776.0 ± 36.7	44.7 ± 2.1
MO BIO - BB	674.6 ± 33.6	32.4 ± 1.6
