# Supplementary material for: Validating DNA Extraction Protocols for Bentonite Clay
Source: mSphere. 2019 Oct 30;4(5):e00334-19. doi: 10.1128/mSphere.00334-19 (PMC6821930; doi:10.1128/mSphere.00334-19)
Supplement: TABLE S2 [file mSphere.00334-19-st002.pdf]

Control	Reads (n)
Extraction kit control	1,585 ± 2,135 (4)
NTC1 (single tube)	73 ± 94 (4)
NTC2 (96-well plate)	1,586 ± 2,728 (6)
