# Supplementary material for: Validating DNA Extraction Protocols for Bentonite Clay
Source: mSphere. 2019 Oct 30;4(5):e00334-19. doi: 10.1128/mSphere.00334-19 (PMC6821930; doi:10.1128/mSphere.00334-19)
Supplement: TEXT S1 [file mSphere.00334-19-s0001.pdf]

DNA extraction from spiked Wyoming MX 80 using a chloroform-based extraction method

DNA extraction from spiked MX-80 samples was performed using a method modified from Lever et al. (2015; from here on referred to as LPII). Wyoming MX-80 bentonite (Caldic Canada; Lot 06525768; Manufactured 2015-Jun-03) was used to prepare a 1:6 slurry (solid:liquid ratio) in sterile distilled and deionized nucleic-acid-free water. MX-80 bentonite slurry was spiked with 14 ng genomic *Escherichia coli* DNA per mg MX-80, mixed by inversion, and incubated at room temperature for 30 min to enable DNA binding to the clay matrix. Six ml spiked MX-80 slurry was added to a 50 ml bead beating tube containing 7 g of 0.1 mm zirconia beads and 2.5 volume extraction buffer LSI (30 mM Tris-HCl, 30 mM EDTA, 800 mM guanidine hydrochloride, 0.5% Triton X-100, pH 8.0) was added. If indicated, phosphate (100 µmol per 1 g MX-80 (dry weight)) was added to LSI. One freeze-thaw cycle was applied before each sample was incubated at 50°C for 1 h. Samples were homogenized for 10 min at 20 Hz in a MM 400 Mixer Mill (Retsch) and then centrifuged at 7,000 g for 10 min at room temperature. Supernatants were mixed with equal volumes of chloroform:isoamyl alcohol (24:1 [vol/vol]). The aqueous phase was retained by centrifugation, and the DNA was precipitated at 4°C overnight with Co-Precipitant Linear Polyacrylamide (4 µl per ml; Bioline), 1.5 volumes of isopropanol and 0.1 volumes of 5 M sodium chloride. The tube was centrifuged at 7,000 g for 30 min. The pellet of DNA was washed with cold 70% ethanol and resuspended in 1 ml PCR water.

In the presence of phosphate, LPII recovered 72% genomic DNA (Figure S4), which is higher than with the PowerSoil Isolation Kit (MO BIO). However, because sample processing time was very high, we used the PowerSoil Isolation Kit for all future extractions because of batch to batch consistency of reagents, fast processing time, circumvention of toxic organic solvents, and scalability for automation of high sample throughput extractions.
